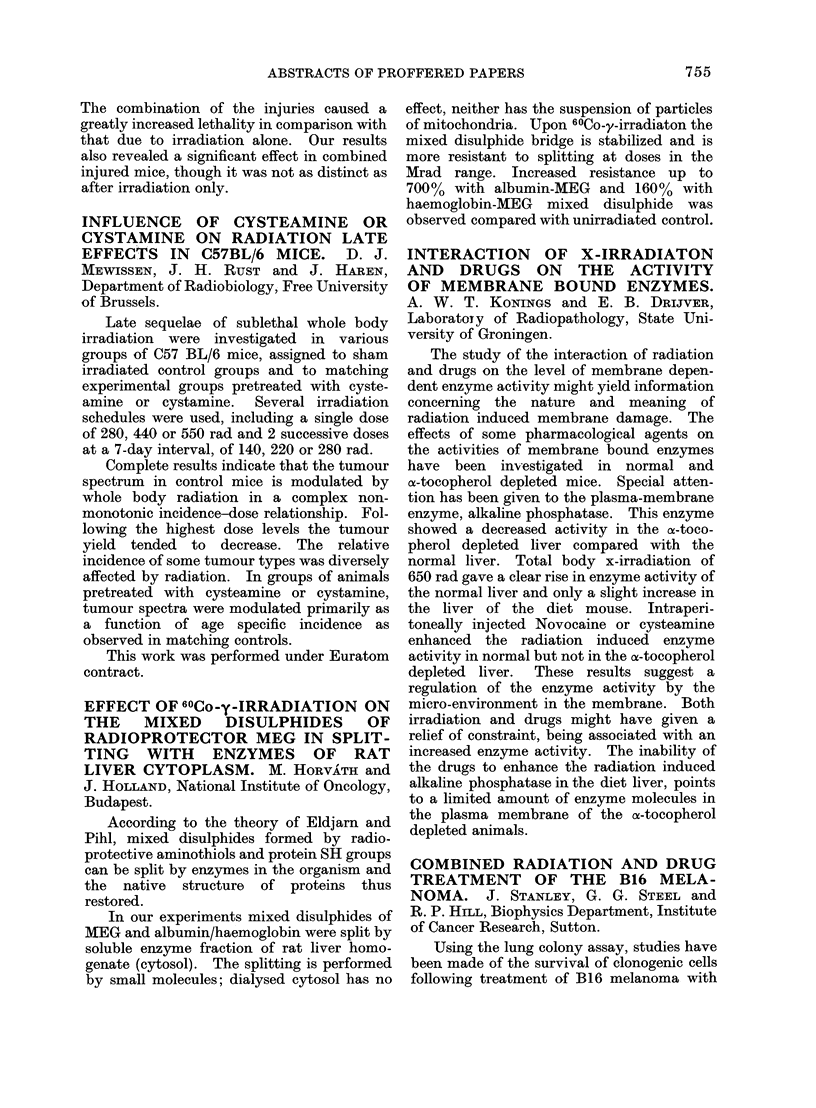# Proceedings: Interaction of X-irradiation and drugs on the activity of membrane bound enzymes.

**DOI:** 10.1038/bjc.1975.302

**Published:** 1975-12

**Authors:** A. W. Konings, E. B. Drijver


**INTERACTION OF X-IRRADIATON AND DRUGS ON THE ACTIVITY OF MEMBRANE BOUND ENZYMES.** A. W. T. Konings and E. B. Drijver, Laboratory of Radiopathology, State University of Groningen.

The study of the interaction of radiation and drugs on the level of membrane dependent enzyme activity might yield information concerning the nature and meaning of radiation induced membrane damage. The effects of some pharmacological agents on the activities of membrane bound enzymes have been investigated in normal and $\alpha$-tocopherol depleted mice. Special attention has been given to the plasma-membrane enzyme, alkaline phosphatase. This enzyme showed a decreased activity in the $\alpha$-tocopherol depleted liver compared with the normal liver. Total body x-irradiation of 650 rad gave a clear rise in enzyme activity of the normal liver and only a slight increase in the liver of the diet mouse. Intraperitoneally injected Novocaine or cysteamine enhanced the radiation induced enzyme activity in normal but not in the $\alpha$-tocopherol depleted liver. These results suggest a regulation of the enzyme activity by the micro-environment in the membrane. Both irradiation and drugs might have given a relief of constraint, being associated with an increased enzyme activity. The inability of the drugs to enhance the radiation induced alkaline phosphatase in the diet liver, points to a limited amount of enzyme molecules in the plasma membrane of the $\alpha$-tocopherol depleted animals.